# Atypical Location of Intracerebral Hemorrhage in Moyamoya Disease

**DOI:** 10.7759/cureus.1948

**Published:** 2017-12-14

**Authors:** Zainah A Abuoliat, Basma A AlFarhan, Aysha A Alshahrani, Amal A AlFarhan, Makki A Almuntashri, Naser Alotaibi

**Affiliations:** 1 College of Medicine, King Saud Bin Abdulaziz University for Health Sciences; 2 Neuroradiology, Medical Imaging Departement, King Abdulaziz Medical City, Ministry of National Guard Health Affairs, Riyadh, Saudi Arabia; 3 Neurology and Internal Medicine Consultant, Stroke and Neurophysiology Specialist, Director of Neurology Residency Training Program, King Abdulaziz Medical City, Ministry of National Guard Health Affairs, Riyadh, Saudi Arabia

**Keywords:** moyamoya disease, cerebral angiography, splenium of corpus callosum hemorrhage

## Abstract

Moyamoya disease is a chronic, progressive bilateral occlusion or stenosis of terminal internal carotid arteries as well as the proximal anterior and middle cerebral arteries. Hemorrhage of the splenium of the corpus callosum rarely occurs with moyamoya disease. In this article, we report a case of a 53-year-old woman diagnosed with moyamoya disease by cerebral angiography. She presented to the emergency department complaining of unsteadiness and a tendency to fall forward for one week. The patient was investigated with head computed tomography (CT) scan upon presentation revealing atypical location of hemorrhage in the corpus callosum, mainly in the splenium.

## Introduction

Moyamoya disease is a chronic, progressive bilateral occlusion or stenosis of terminal internal carotid arteries as well as the proximal anterior and middle cerebral arteries [1-2]. “Moyamoya” in Japanese means appearing as a “puff of smoke” and this is due to the angiographic appearance of collateral vascularization at the base of the brain from the compensatory dilatation of lenticulostriate and thalamostriate arteries [3]. Moyamoya is divided into two entities: patients with distinctive cerebrovasculopathy of moyamoya who also have well-known associated conditions such as sickle-cell disease and Down syndrome are classified as having moyamoya syndrome, while patients with no associated risk factors are identified as having moyamoya disease [4]. When the term moyamoya is referred to alone, it indicates the distinctive findings of collateral  developments and dilatation of vessels on angiography [5]. The common presentation of moyamoya in children is ischemic stroke, while adults commonly present with subarachnoid or intracerebral hemorrhage most commonly involving the frontal horn of lateral ventricle, basal ganglia, and thalamus [3]. Intracerebral hemorrhage is a common presentation of moyamoya disease. However, splenium hemorrhage is rare. This is a case of atypical presentation of moyamoya disease as a splenium hemorrhage.

## Case presentation

**Figure 1:**
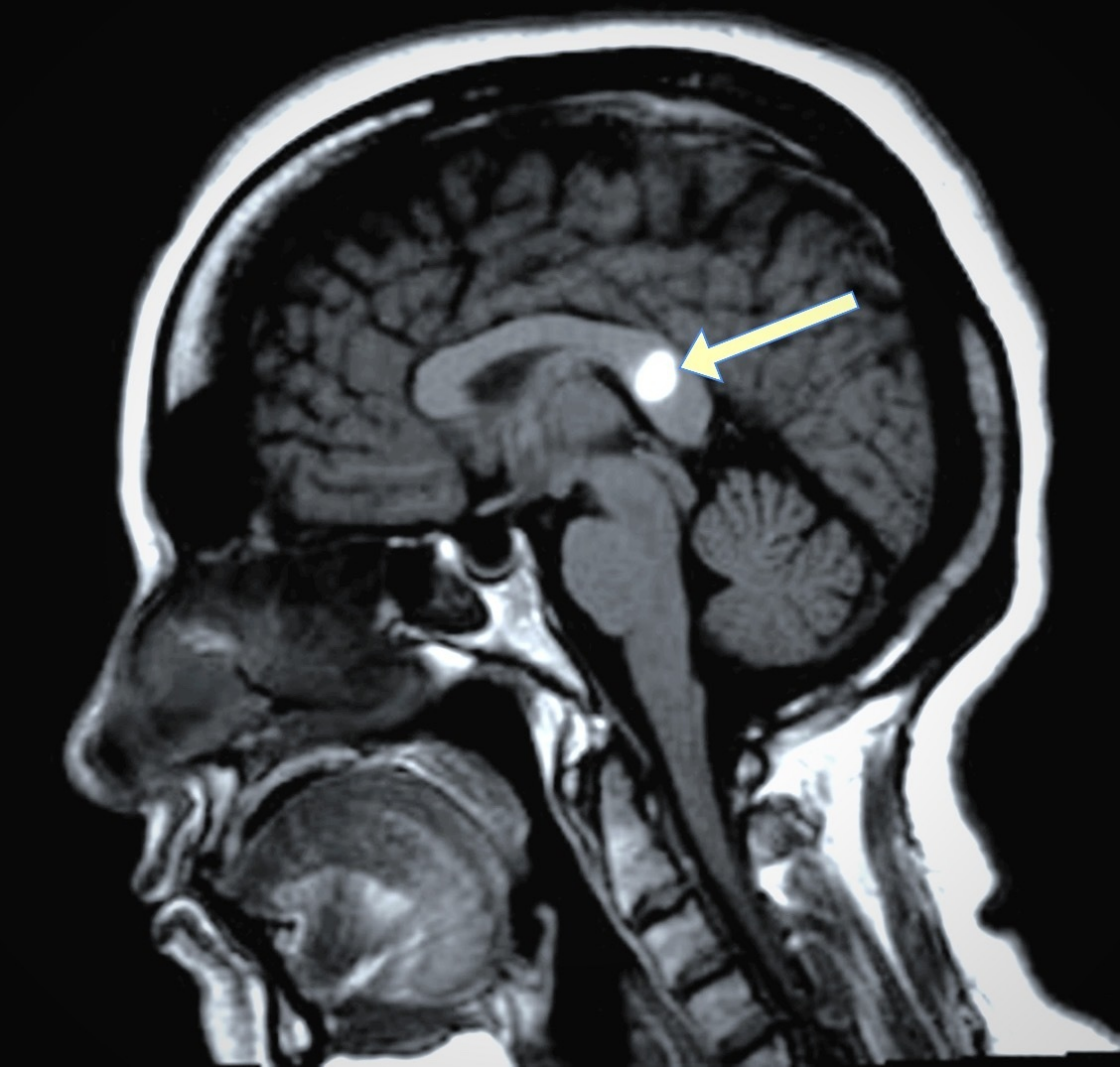
Brain magnetic resonance image (MRI T1W1) showing subacute hematoma of splenium of corpus callosum

A 53-year-old Arab woman living in Saudi Arabia presented to the emergency department with an acute onset of unsteadiness, a tendency to fall forward present for one week, and slurred speech present for one day. She had a negative history of loss of consciousness, abnormal movement, weakness, numbness, and vertigo. She had a history of chronic headaches. She has diabetes and suffered a stroke two years ago. She had one abortion and twelve successful pregnancies; the last two were C-section. She had no history of sickle-cell disease or other chronic diseases. Her neurological assessment was normal with no focal signs. Other examinations were unremarkable. A head computed tomography (CT) scan and a magnetic resonance imaging (MRI) of her brain (Figure 1) upon presentation revealed acute corpus callosum hemorrhage.

**Figure 2:**
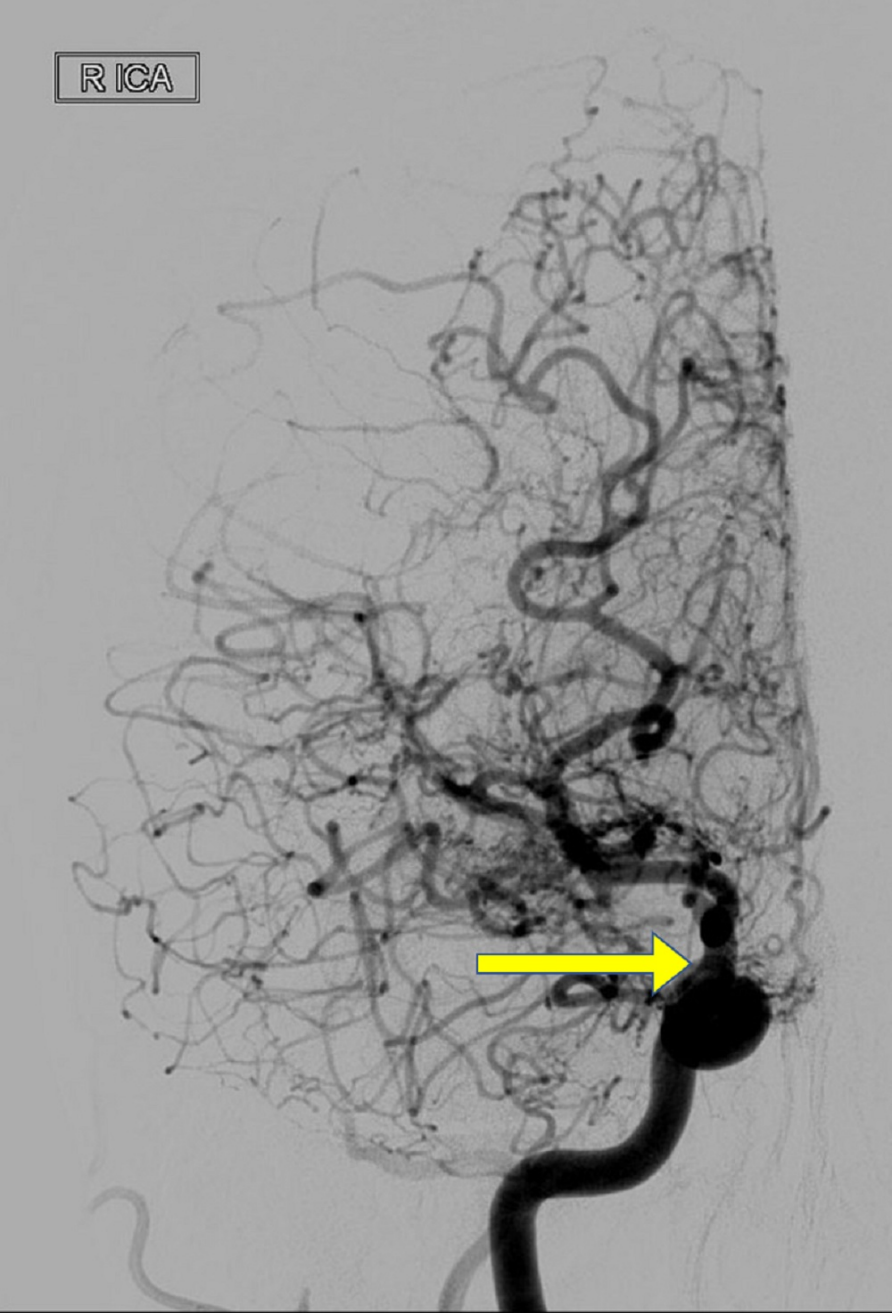
Cerebral angiogram showing severe narrowing of the right internal carotid arteries of the middle cerebral artery with multiple surrounding collateral arteries

**Figure 3:**
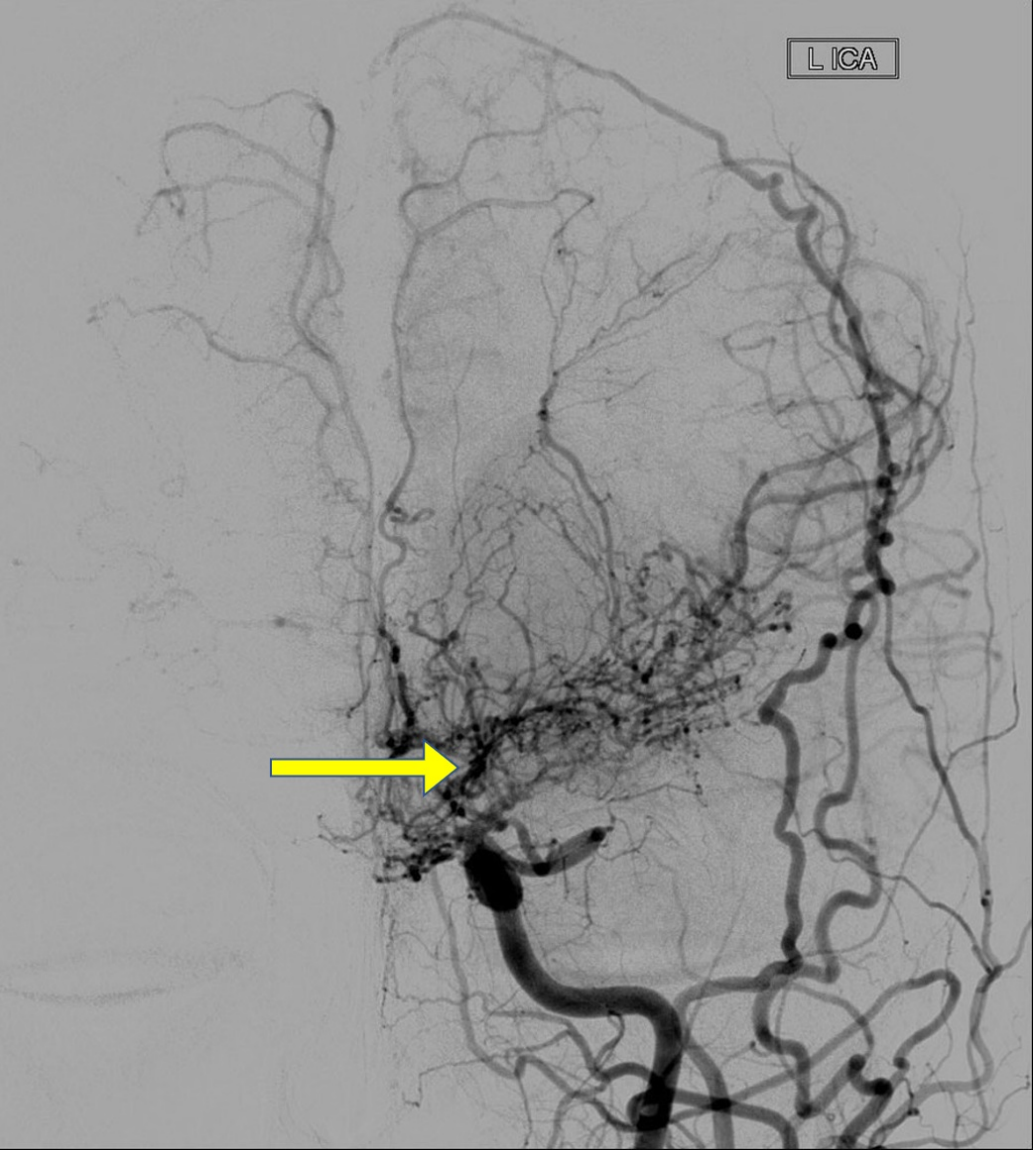
Cerebral angiogram wherein the left internal carotid artery (ICA) shows occlusion of the distal ICA of main left middle cerebral artery with stenosis, with multiple collaterals around the occluded vessels displaying the moyamoya phenomena

Her brain CT venogram was normal. Other diagnostic studies were normal. A cerebral angiogram (Figures 2-3) showed severe narrowing or occlusion of both distal internal carotid arteries (ICA) with multiple intracranial collaterals, including the choroidal arteries and the posterior pericallosal artery as well as the lenticulostriate arteries; this was a classical presentation of the moyamoya pattern.

## Discussion

Takeuchi and Shimizu first described Moyamoya disease in Japan in 1957 [2]. Moyamoya was more prevalent in the Asian population and is the most common cerebrovascular disease in Japanese children with a prevalence rate of 0.003 [6]. Moyamoya was originally considered to affect individuals of Asian heritage predominantly but has now been observed throughout the world [1]. However, there were no studies about the prevalence of moyamoya disease in Arab ethnic groups, particularly in Saudi Arabia. Bimodal distribution of moyamoya was observed with two peaks for age between five to nine and 45 to 59 years [6]. A 1.8:1 female to male ratio was noted, and 10% of the cases had a positive family history [7].

Moyamoya is typically accompanied with persistent headaches refractory to medical therapies. The presentation of moyamoya disease differs substantially between children and adults. Children usually present with brain infarct while adults typically have hemorrhagic events. The location of the intracerebral hemorrhage is commonly observed to be in the frontal horn of the lateral ventricle, basal ganglia, and thalamus. Ischemic events were the most common presentations in both children and adults according to Chiu, et al. [3]. Hemorrhage in the splenium of the corpus callosum is rare; head trauma, neoplasm, aneurysms of the pericallosal artery, and arteriovenous malformation are considered as typical causes of corpus callosum hemorrhage. Moyamoya disease is rarely implicated as a causative factor of corpus callosum hemorrhage and involvement of corpus callous can only be explained by the underlying pathology of moyamoya diseases. The rupture of the abnormally dilated collateral vessels (moyamoya disease) caused the splenium hemorrhage. Moreover, intracranial hemorrhage in patients with moyamoya disease is extremely rare unless there is a clear cerebral aneurysm on angiography. Intracranial hemorrhage is most commonly due to rupture of fragile vessels caused by moyamoya disease [8]. There was one case in the literature review similar to our case. They reported corpus callosum hemorrhage in a 31-year-old woman diagnosed with moyamoya disease. Her radiological findings strongly suggest that splenial hemorrhage happened due to the rupture of the dilated abnormal collateral vessels that originated from the medial posterior choroidal artery penetrating the corpus callosum [4].

Another study found two cases of moyamoya disease with a small hemorrhage in the splenium. Similarly, these cases had marked enlargement of the choroidal and posterior pericallosal arteries [9]. The process of narrowing of cerebral vessels is the core of the pathophysiology of moyamoya and seems to be associated with several conditions such as sickle-cell anemia, neurofibromatosis-1, trisomy 21, congenital heart defects, antiphospholipid syndrome, and renal artery stenosis. However, more than half of the adults seen with this disease have no cause for their moyamoya syndrome [7]. The diagnosis of moyamoya diseases is made by cerebral angiography. The cerebral angiography shows a characteristic finding of moyamoya such as unilateral/bilateral occlusion or stenosis of terminal internal carotid arteries and the proximal anterior and middle cerebral arteries in the basal ganglia [10]. The gold standard of diagnosis is cerebral angiography, but MRI and magnetic resonance angiography (MRA) are also acceptable methods of diagnosis [3]. The Research Committee on Spontaneous Occlusion of the Circle of Willis (Moyamoya Disease) of the Japan Ministry of Health defined the diagnostic criteria of moyamoya disease as bilateral stenosis of the terminal portion of the internal carotid artery with or without the proximal portion of the anterior and/or the middle cerebral arteries. These findings described by the Research Committee on Spontaneous Occlusion of the Circle of Willis should be observed in the absence of arteriosclerosis, autoimmune disease, brain neoplasm, Down syndrome, head irradiation, head trauma, meningitis, neurofibromatosis, and sickle cell anemia [3].

There is no known curative treatment for moyamoya disease. Acute management is mainly symptomatic and directed towards reducing elevated intracranial pressure, improving cerebral blood flow, and controlling seizures [3]. Antihypertensive, calcium channel blockers, and daily aspirin are used as needed to maintain constant low blood pressure.

Surgical revascularization is thought to improve cerebral perfusion and to reduce the risk of subsequent stroke in both pediatric and adult patients [7]. Chiu, et al. compared 20 patients who had surgical revascularization with 15 patients who were treated medically and found no statistically significant difference in the five-year stroke recurrence rate between the two groups [3].

## Conclusions

Although isolated splenic hemorrhage is rare, it is a recognized manifestation of moyamoya disease. Therefore, intracranial vascular imaging should be done to exclude moyamoya disease. As current treatment is solely supportive, further research is needed.
